# Association between systemic zinc and oxidative stress levels and periodontal inflamed surface area

**DOI:** 10.55730/1300-0144.5868

**Published:** 2024-07-07

**Authors:** Ayşegül SARI, Serdar DOĞAN, Luigi NIBALI

**Affiliations:** 1 Department of Periodontology, Faculty of Dentistry, Hatay Mustafa Kemal University, Hatay, Turkiye; 2 Periodontology Unit, Centre for Host-Microbiome Interactions, King’s College London Dental Institute, London, UK; 3 Department of Medical Biochemistry, Faculty of Medicine, Hatay Mustafa Kemal University, Hatay, Turkiye

**Keywords:** Periodontal disease, periodontal inflamed surface area, zinc, oxidative stress

## Abstract

**Background/aim:**

Zinc is a structural component of some enzymes. The aim of this study was to evaluate the potential associations between serum zinc and oxidative stress levels and periodontal inflamed surface area (PISA).

**Materials and methods:**

This study included 90 patients divided into three groups: a periodontitis group (P; n = 30), a gingivitis group (G; n = 30), and a periodontal health group (PH; n = 30). Periodontal parameters were recorded and PISA values were calculated. Serum total antioxidant status (TAS), total oxidant status (TOS), and zinc levels were examined biochemically. Oxidative stress index (OSI) levels were calculated.

**Results:**

All clinical periodontal parameters, periodontal epithelium surface area, and PISA values were lower in the PH group than the P and G groups (p < 0.05). Serum zinc and TAS values were higher in the PH group than the P and G groups (p < 0.05). Serum TOS and OSI values were lower in the PH group than the G and P groups (p < 0.05). Serum OSI levels were lower in the G group than the P group (p < 0.05). PISA was associated with serum zinc (β = −28.96, 95% CI = (−38.95, −18.98), p < 0.001) and OSI (β = 89.84, 95% CI = (20.63, 159.05), p = 0.011) levels in the multivariate generalized linear model.

**Conclusion:**

PISA values were associated with decreasing serum zinc and TAS and increased TOS and OSI levels. Zinc deficiency can be associated with the severity of periodontal disease and higher oxidative stress levels.

## 1. Introduction

Periodontal diseases are common inflammatory diseases that occur as a result of disruption of the homeostasis between oral pathogens and the host [1]. Periodontopathogens induce a host inflammatory response that includes the release of reactive oxygen species (ROS) that can cause periodontal tissue destruction [2]. Systemic inflammatory markers can be associated with the amount of periodontal inflamed tissue [3]. Periodontal inflamed surface area (PISA) is calculated by measuring the surface area of the bleeding pocket epithelium in square millimeters [4]. It has been used as an indicator of periodontal inflammation as it can quantify the inflammatory burden caused by periodontal disease [5].

ROS plays an important role in antimicrobial defense, gene regulation, and cell signaling [6]. An excess of ROS leads to increased oxidant load with unchanged or reduced antioxidant capacity and causes oxidative stress in tissues. Oxidative stress damages biomolecules and cell membranes inside the cell. Intracellularly, ROS damage biomolecules and cell membranes [7]. During phagocytosis in periodontal disease pathogenesis, free radicals, which are the end products of mitochondrial respiratory burst in polymorphonuclear neutrophils, damage lipid peroxidation, proteins, and DNA [8]. This mechanism leads to an oxidative imbalance that triggers proinflammatory pathways and osteoclastogenesis, and, thus, the bone loss observed in patients with periodontitis [9]. In addition, ROS is responsible for the loss of attachment leading to periodontal destruction by directly damaging extracellular connective tissue [10]. Several studies revealed increased levels of oxidative stress in patients with periodontitis, further strengthening the association between periodontal inflammation and oxidative stress [11–13].

Periodontal tissue viability is closely related to some micronutrients such as zinc (Zn) [14]. Zinc is a structural component of some enzymes as a cofactor, such as copper-zinc superoxide dismutase and superoxide dismutase 1 [15]. It has antioxidant properties [14]. Changes in zinc levels can cause oxidative stress and neutralize bacterial toxins [16]. It plays a role in cell division, DNA synthesis, immune metabolism, and wound healing [14], and it is essential in maintaining the structural integrity of proteins and protein folding [17]. It also affects T-lymphocyte activation and development [17]. Two studies showed that serum zinc levels decrease in periodontal disease [18,19]. Another study noted that reduced zinc levels were associated with periodontal bone loss [20]. On the other hand, some studies have suggested that oxidative stress can increase intracellular zinc levels [21,22]. A recent study reported that periodontal disease progression can be affected by increasing intracellular zinc concentration through an oxidative stress signaling pathway in periodontal epithelial cells [23].

It is not clear whether periodontal inflammation measured as PISA affects serum zinc and serum oxidative stress levels. Therefore, the aim of this study was to evaluate a potential association between serum zinc and oxidative stress levels and PISA. Our null hypothesis was that there are no differences in serum zinc levels and total oxidant status (TOS), total antioxidant status (TAS), and oxidative stress index (OSI) in patients with periodontitis and gingivitis and periodontally healthy individuals.

## 2. Materials and methods

### 2.1. Study population

A cross-sectional comparative study was carried out as a joint collaboration between the Department of Periodontology in the Faculty of Dentistry and the Department of Biochemistry in the Faculty of Medicine at Hatay Mustafa Kemal University in Türkiye. The study protocol was approved by the Ethics Committee for the Use of Human Subjects in Research of Hatay Mustafa Kemal University (Protocol No: 2021/174) and was carried out in accordance with the tenets of the Declaration of Helsinki. Individuals were included in the study from December 2021 through February 2022. Written consent forms were obtained from all patients before their participation in the study [24].

All patients included were from the Department of Periodontology in the Faculty of Dentistry. The patients were divided into periodontitis and gingivitis groups according to their periodontal status. Periodontally healthy staff and students from the Faculty of Dentistry and the Department of Biochemistry in the Faculty of Medicine were included in the periodontally healthy group. A total of 125 individuals were examined for this cross-sectional comparative study. Thirty-five individuals were not included as they did not meet the inclusion criteria, while the remaining 90, including three groups with periodontitis (P; 30 patients), gingivitis (G; 30 patients), and periodontal health (PH; 30 participants) were included.

The inclusion criteria were as follows: i) individuals who had never smoked or had quit smoking at least 6 years before; ii) no history of periodontal treatment in the past 6 months; iii) no antibiotic therapy in the past 3 months; iv) more than 20 teeth present.

The exclusion criteria included the following: i) pregnancy; ii) systemic diseases or conditions (cardiovascular diseases, rheumatoid arthritis, diabetes, AIDS, etc.); iv) BMI of ≥25 kg/m^2^ (calculated according to self-reported height and weight).

### 2.2. Clinical periodontal parameters

Periodontal clinical parameters were recorded by a single calibrated examiner (A.S.; intraclass correlation coefficient = 0.95). Clinical attachment level (CAL) values were used for the determination of intraexaminer agreement. Reproducibility was calculated according to repeated examinations for 8 subjects at a 1-h interval. Clinical periodontal measurements were assessed using the following periodontal measurements for periodontal diagnosis.

Measurements were performed using a Williams periodontal probe (Hu-Friedy, Chicago, IL, USA) and included probing pocket depth (PPD), CAL, plaque index (PI) [25], gingival index (GI) [26], and percentage of bleeding on probing (BOP) [27] at six sites per tooth (mesiobuccal, buccal, distobuccal, mesiolingual, lingual, and distolingual) on each tooth.

Diagnosis of periodontal diseases and conditions was performed according to the radiographic and clinical diagnostic criteria proposed by the 2017 World Workshop on Classification of Periodontal and Peri-implant Diseases and Conditions [28]. Individuals with a BOP of <10% without attachment loss or radiographic bone loss were considered to have periodontal health [29]. Only generalized gingivitis patients were included in this study. Individuals presenting with a BOP of ≥30% and PPD of ≤3 mm without radiographic bone loss or attachment loss were considered to have gingivitis [30]. Only stage III–IV (severe) periodontitis was included in the present study. The criteria for periodontitis included patients with CAL of ≥5 mm in two or more interproximal sites and PPD of ≥6 mm in one or more interproximal sites [31].

The periodontal epithelium surface area (PESA, mm^2^) and PISA as its derived measure were estimated using the average surface area of each tooth type along with periodontal measures using a freely accessible Excel spreadsheet (https://www.parsprototo.info). PPD and BOP values for six sites per tooth were entered in this spreadsheet to estimate PISA values [5].

### 2.3. Collection of blood samples

Peripheral venous blood samples were taken from the patients. The serum was separated from the cells by centrifugation at 3000 rpm for 10 min, after which it was stored at −80 °C until biochemical analysis was performed.

### 2.4. Laboratory analyses

Laboratory analyses were performed by a single biochemist (S.D.) blinded to the clinical status of the participants.

#### 2.4.1. Measurement of zinc levels in serum samples

Serum zinc levels were measured by a colorimetric method using an Archem zinc kit (Archem, İstanbul, Türkiye) with a Siemens Atellica autoanalyzer (Siemens, Munich, Germany). Zinc results were given as μg/dL. Quality controls of the analysis were conducted routinely.

#### 2.4.2. Measurement of oxidative stress levels in serum samples

Serum total oxidant status (TOS) and total antioxidant status (TAS) levels were determined by the colorimetric method described by Erel (Rel Assay Diagnostics, Ankara, Türkiye) [32,33]. TAS results were expressed as mmol Trolox equiv./L and the results of TOS were given as μmol H_2_O_2_ equivalent per liter (μmol H_2_O_2_ equiv./L). The oxidative stress index (OSI) was calculated as follows: OSI (arbitrary unit) = TOS (μmol H_2_O_2_ equiv./L)/TAS (μmol Trolox equiv./L) × 100 [34].

### 2.5. Statistical analysis

The primary outcome of the study was the difference in serum zinc levels by periodontal status. The secondary outcome was the relationship among serum zinc, oxidative stress levels, and PISA. The 90 participants were selected as a convenience sample, as there were no previously published data on serum zinc levels in health versus gingivitis versus periodontitis. A post hoc power calculation was performed based on zinc levels in four groups using G*Power software version 3.1.9.7 (University of Dusseldorf, Dusseldorf, Germany). This analysis revealed that 30 patients per group would provide 100% power for an effect size f of 0.69 (standard deviation: 14.27) with α = 0.05.

The normal distribution of continuous variables was evaluated by Shapiro–Wilk test. Nonparametric statistical methods were performed for values with skewed distribution. Descriptive statistics were presented as median (interquartile range) for the nonnormally distributed variables and as mean and standard deviation for normally distributed variables. One-way analysis of variance (ANOVA) was performed for the comparison of more than two normally distributed groups and the Tukey test was performed for post hoc pairwise multiple comparison analyses. The Kruskal–Wallis test was performed for the comparison of more than two nonnormally distributed variables. For pairwise post hoc comparisons, the Bonferroni-corrected Mann–Whitney U test was performed. The chi-square test was used to analyze associations between categorical variables. The correlation between two nonnormally distributed variables was evaluated by Spearman rho correlation coefficient analysis. A multivariate general linear model was established to evaluate factors affecting PISA values.

## 3. Results

### 3.1. Demographic findings

Table 1 shows the demographic characteristics of the groups. The demographic variables were similar between the group (p > 0.05).

### 3.2. Clinical findings

Table 2 shows the periodontal parameters. All clinical periodontal parameters, PESA, and PISA values were lower in the PH group than the P and G groups (p < 0.05). Furthermore, PPD and PESA values were lower in the G group than the P group (p < 0.05).

### 3.3. Laboratory findings

Table 3 shows the intergroup biochemical parameters of the groups. Serum zinc and TAS values were higher in the PH group than the P and G groups (p < 0.05). Serum TOS and OSI values were lower in the PH group than the G and P groups (p < 0.05). Serum OSI were lower in the G group than the P group (p < 0.05).

### 3.4. Correlations

Table 4 shows correlations between clinical periodontal parameters and serum laboratory parameters. Negative correlations were detected between all clinical periodontal parameters, PESA, and PISA values and serum zinc and TAS values. Positive correlations were detected between all clinical periodontal parameters, PESA, and PISA values and serum TOS and OSI values.

Table 5 shows correlations between serum TAS, TOS, and OSI values and zinc levels. Positive correlations were detected between serum TAS and zinc levels (p < 0.05). Negative correlations were detected between serum TOS and OSI and zinc levels (p < 0.05).

Table 6 shows the results of general linear regression analyses of factors affecting PISA levels. Increasing PISA values were associated with decreasing serum zinc (β = −28.96, 95% CI = (−38.95, −18.98), p < 0.001) and increasing OSI (β = 89.84, 95% CI = (20.63, 159.05), p = 0.011) values in the multivariate generalized linear model with independent confounding factors of age, sex, and BMI.

## 4. Discussion

To the best of the authors’ knowledge, this is the first study to evaluate serum zinc and oxidative stress levels in periodontitis, gingivitis, and periodontal health. The findings of this study showed that serum zinc and oxidative stress levels were higher in periodontitis and gingivitis. There were significant associations between serum zinc and oxidative stress levels and PISA.

Zinc has an essential role in antioxidant functions, gene expression, cell proliferation and diffraction, wound healing, and the stabilization and integrity of biomembranes [35]. Zinc deficiency can increase proinflammatory cytokine levels and affect the functioning of immune cells such as lymphocytes, natural killer cells, T cells, neutrophils, and monocytes [36]. Decreased zinc levels can play a role in periodontal bone loss [20]. Significantly increased serum zinc levels were reported in the periodontitis and gingivitis groups compared with the periodontally healthy group in this study. To the best of the authors’ knowledge, this is the first study to compare patients with gingivitis to periodontally healthy controls according to serum zinc levels. However, a previous study that evaluated zinc levels in patients with and without diabetes and with periodontitis or periodontal health showed that zinc levels were lower in patients with periodontitis with and without diabetes than in periodontally healthy controls [19]. Another nonsurgical periodontal treatment follow-up study in patients with controlled or uncontrolled diabetes found that serum zinc concentrations were increased in both periodontitis groups [37]. The results of the present study were compatible with those previous studies, suggesting decreased zinc levels in periodontal disease [19,37]. According to these results, it can be suggested that zinc concentrations can contribute to periodontal disease progression.

Periodontal diseases have higher levels of inflammation and oxidative stress compared to periodontal health [2]. Oxidative stress is an important parameter in periodontal disease pathophysiology [8]. Besides measuring oxidative and antioxidative parameters separately, the measurability of TAS and TOS presents a practical advantage [32,33]. In addition, OSI is an important indicator of the change in the balance of oxidation and antioxidation in the body [34]. Significantly decreased serum TAS and increased TOS and OSI levels were reported in the periodontitis and gingivitis groups compared to the periodontally healthy group in this study. This result was in agreement with the previous literature [11–13]. There are various inflammatory pathway hypotheses that reveal the relationship between oxidative stress and zinc [38,39].

Zinc is associated with some enzyme activities that are combated by oxidative stress, such as superoxide dismutase (SOD) activity [38]. It also protects biological structures from oxidative stress by affecting the metal ions involved in the formation of hydroxyl radicals [39]. The present study showed that there were positive correlations between serum TAS and zinc levels. Also, negative correlations were detected between both serum TOS and OSI and zinc levels. An animal study reported that a zinc-deficient diet increased oxidative stress in vascular smooth muscle cells [40]. A placebo-controlled human study reported that zinc supplementation significantly reduced plasma lipid peroxidation markers in the study group compared to the placebo group [41]. The findings of the present study are compatible with those of previous studies [40,41]. A review article suggested that zinc, being related to increased oxidative stress and decreased antioxidants, has potential diagnostic and prognostic implications [42]. It can also suggest that serum zinc levels can affect periodontal disease progression by association with oxidative stress. On the other hand, zinc can upregulate NF-κB transcriptional activity by increasing the affinity between NF-κB and DNA [43]. In this case, oxidative stress-induced increases in intracellular zinc may act as a “Zn wave” increasing the binding between DNA and NF-κB [44]. One previous study showed that oxidative stress promoted the release of Zn^2+^ from intracellular nonproteinic thiols and also increased the permeability of the cell membrane to Zn^2+^, leading to an influx of Zn^2+^ from the extracellular space [23]. The results of these studies suggest that oxidative stress increases in parallel with the zinc level in the intrinsic area. These results conflict with the findings of the present study in terms of serum levels of zinc, which is present in the structure of many antioxidant enzymes. It can be suggested that zinc and oxidative stress have a different and bidirectional relationship in intrinsic and extrinsic areas.

PISA represents a quantification of the inflammatory burden of the periodontium [5] and is often used for determining dose–response relationships on the periodontal–systemic axis [11]. Findings from the present study showed that PISA had negative correlations with serum zinc and TAS values and positive correlations with TOS and OSI values. PISA was associated with serum zinc and OSI levels in the multivariate generalized linear model after adjusting for the confounders which were age, sex, and BMI. Other important factors to adjust for are diet and possibly socioeconomic factors/lifestyle, as they may also be related to zinc and oxidative stress levels. The fact that these data were not recorded is among the limitations of this study. A one-unit increase in PISA was associated with a decrease in serum zinc level of approximately 28 units (β = −28.96, 95% CI = (−38.95, −18.98), p < 0.001) and an increase in OSI of approximately 90 units (β = 89.84, 95% CI = (20.63, 159.05), p = 0.011) in the present study. A previous study reported positive correlations between PISA and TOS and OSI values. PISA was associated with serum OSI level in the same study (β = 0.0001, 95% CI = (0.00008, 0.001)) [11]. The fact that a decrease in serum zinc level is associated with an increase in PISA value may suggest that zinc deficiency is associated with the severity of periodontal disease. There is no previous study in the literature that evaluated zinc levels and PISA values. The PISA values associated with decreasing serum zinc levels may suggest that zinc deficiency is associated with the severity of periodontal disease. Metabolic changes in serum Zn levels and increased levels of oxidative stress may contribute to the development of periodontal disease or disease progression, leading to decreased regenerative capacity and impaired immune function. The findings of the present study may also suggest that zinc-rich dietary ingredients or nutritional supplements may reduce periodontal disease progression and increase periodontal therapy success. A review article evaluating the effect of micronutrient malnutrition on periodontal disease concluded that deficiencies of micronutrients such as zinc, magnesium, and vitamin D can affect bone mineralization, collagen structure, inflammation, and oxidative stress [14]. In another cross-sectional study involving 60 patients, those with diabetes mellitus and periodontitis had lower levels of vitamin C and zinc than healthy individuals. That study suggested that micronutrient supplementation in vitamin-deficient patients can present an advantage in preventing periodontal disease [19]. It has been shown that treatments that reduce calorie intake and only support certain proteins and vitamins, such as diets that mimic fasting, change the immune response [45]. The findings of the present study are consistent with those of previous studies [14,19,45]. Further randomized controlled studies are needed to clarify the relationship between periodontal disease and serum zinc levels.

Limitations of this study were the absence of variables such as diet and socioeconomic factors/lifestyle in the adjusted analysis and the cross-sectional design, which cannot account for potential causality.

Within these limitations, this study suggests that periodontal diseases may be associated with systemic zinc and oxidative stress levels.

## Figures and Tables

**Table 1:** Characteristics of the study population.

Variable	PH group (n=30)	G group (n=30)	P group (n=30)	p-value*
**Age (IQR: 25–75)**	38 (22–46)	31 (19–52)	40 (18–45)	0.964
**Sex (male/female) n (%)**	12/18	13/17	13/17	0.955
**BMI (kg/m** **^2^** **) (IQR: 25–75)**	23.7 (18.5–24.6)	23.2 (17.9–24.6)	24.1 (17.3–24.4)	0.963

* p-values obtained from the Kruskal–Wallis test and chi-squared test.

Data are expressed as median, 25% to 75%, and n (%). Statistically significant at p < 0.05.

PH: periodontally healthy, G: gingivitis, P: periodontitis.

BMI: body mass index.

**Table 2:** Comparison of clinical periodontal parameters among all groups.

Variable	PH group (n=30)	G group (n=30)	P group (n=30)	p-value
**PI**	0.05 (0–0.25)	2 (0–2.1) †	2.45 (0.5–3) †	<0.001*
**GI**	0.2 (0–0.3)	1.9 (1.4–2.5) †	2.7 (1.–2.9) †	<0.001*
**BOP (%)**	2.7 (0–5.4)	91 (44.6–100) †	96.3 (42.7–100) †	<0.001*
**PPD (mm)**	1.5±0.2	3.2±0.5 †	4.3±0.8 †, ‡	<0.001**
**CAL (mm)**	1.5±0.2	2.9±0.2 †	4.6±0.9 †	<0.001**
**Missing teeth number**	0±0.8	1±2.3 †	2±3.5 †	0.001**
**PESA**	770.5 (10.2–852.8)	1594.1 (1120.1–1954) †	2174.8 (983.6–2683.7) †, ‡	<0.001*
**PISA**	21.2 (0–41.1)	1530.4 (529.8–1951.7) †	2081.8 (739.4–2630.3) †	<0.001*

* p-values obtained from the Kruskal–Wallis test for nonparametric variables.

** p-values obtained from ANOVA test and Tukey test for parametric variables.

Significance values adjusted by the Bonferroni correction for multiple tests.

Data are expressed as median and 25% to 75% and mean ± SD.

Statistically significant at p < 0.05,

† p < 0.05 versus PH,

‡ p < 0.05 versus G.

PH: periodontally healthy, G: gingivitis, P: periodontitis.

PI: plaque index, GI: gingival index, BOP: percentage bleeding on probing, PPD: probing pocket depth, CAL: clinical attachment level, PESA: periodontal epithelial surface area, PISA: periodontal inflamed surface area.

**Table 3:** Comparison of biochemical markers among all groups.

Variable	PH group (n=30)	G group (n=30)	P group (n=30)	p
**Zinc μg/dL**	91 (62–102)	77 (57–86.5) †	69 (54–80.3) †	<0.001*
**TAS (mmol Trolox Eq/L)**	2.18 (1.85–2.24)	1.96 (1.46–2.09) †	1.87 (1.31–2) †	<0.001*
**TOS (μmol H** **_2_** **O** **_2_** ** Eq/L)**	10.34±1.65	13.25±2.24 †	14.65±2.46 †	<0.001**
**OSI (arbitrary unit)**	0.48 (0.33–0.51)	0.67 (0.48–0.75) †	0.84 (0.53–0.99) †, ‡	<0.001*

* p-values obtained from the Kruskal–Wallis test for nonparametric variables.

** p-values obtained from ANOVA test and Tukey test for parametric variables.

Significance values adjusted by the Bonferroni correction for multiple tests.

Data are expressed as median and 25% to 75% and mean ± SD.

Statistically significant at p < 0.05,

† versus PH,

‡ versus G.

PH: periodontally healthy, G: gingivitis, P: periodontitis.

TAS: total antioxidant status, TOS: total oxidant status, OSI: oxidative stress index.

**Table 4:** Correlations between clinical periodontal parameters and zinc, TAS, TOS, and OSI levels.

Variable		Zinc μg/dL	TAS (mmol Trolox Eq/L)	TOS (μmol H_2_O_2_ Eq/L)	OSI (arbitrary unit)
**PI**	r	−0.430*	−0.625*	0.474*	0.685*
	p	<0.001	<0.001	<0.001	<0.001
**GI**	r	−0.427*	−0.554*	0.523*	0.693*
	p	<0.001	<0.001	<0.001	<0.001
**BOP (%)**	r	−0.480*	−0.486*	0.467*	0.620*
	p	<0.001	<0.001	<0.001	<0.001
**PPD**	r	−0.516*	−0.560*	0.591*	0.732*
	p	<0.001	<0.001	<0.001	<0.001
**CAL**	r	−0.531*	−0.559*	0.610*	0.757*
	p	<0.001	<0.001	<0.001	<0.001
**Missing teeth number**	r	−0.279*	−0.181	0.303*	0.332*
	p	0.008	0.090	0.004	0.002
**PESA**	r	−0.533*	−0.514*	0.607*	0.715*
	p	<0.001	<0.001	<0.001	<0.001
**PISA**	r	−0.549*	−0.522*	0.550*	0.694*
	p	<0.001	<0.001	<0.001	<0.001

* Correlation is significant at the 0.01 level.

Spearman’s rank correlation coefficient.

PI: plaque index, GI: gingival index, BOP: percentage bleeding on probing, PPD: probing pocket depth, CAL: clinical attachment level.

PESA: periodontal epithelial surface area, PISA: periodontal inflamed surface area.

TAS: total antioxidant status, TOS: total oxidant status, OSI: oxidative stress index.

**Table 5:** Correlations between serum zinc and TAS, TOS, and OSI levels.

Variable		TAS (mmol Trolox Eq/L)	TOS (μmol H_2_O_2_ Eq/L)	OSI (arbitrary unit)
**Zinc μg/dL**	r	0.425*	−0.466*	−0.530*
	p	<0.001	<0.001	<0.001

* Correlation is significant at the 0.01 level.

Spearman’s rank correlation coefficient.

TAS: total antioxidant status, TOS: total oxidant status, OSI: oxidative stress index.

**Table 6:** General linear regression analysis of factors affecting PISA levels.

Variables	Univariate generalized linear model		Multivariate generalized linear model	
	
	Coefficient (95% CI)	p-value	Coefficient (95% CI)	p-value

**Age**	3.65 (−15.66, 22.98)	0.704		

**BMI**	9.21 (−103.24, 84.82)	0.844		

**ZINC**	−30.47 (−41, −19.95)	<0.001	−28.96 (−38.95, −18.98)	<0.001

**OSI**	113.31 (31.15, 195.48)	0.006	89.84 (20.63, 159.05)	0.011

**Sex N (%)**	7.11 (−429.26, 443.46)	0.974		
**Female Male**	1 (Reference)			

Statistically significant at p < 0.05.

PISA: periodontal inflamed surface area, BMI: body mass index, OSI: oxidative stress index.
